# AgeGuess, a Methylomic Prediction Model for Human Ages

**DOI:** 10.3389/fbioe.2020.00080

**Published:** 2020-03-10

**Authors:** Xiaoqian Gao, Shuai Liu, Haoqiu Song, Xin Feng, Meiyu Duan, Lan Huang, Fengfeng Zhou

**Affiliations:** ^1^BioKnow Health Informatics Laboratory Key Laboratory of Symbolic Computation and Knowledge Engineering, College of Computer Science and Technology, Ministry of Education, Jilin University, Changchun, China; ^2^College of Computer Science, Hubei University of Technology, Wuhan, China; ^3^Key Laboratory of Symbolic Computation and Knowledge Engineering, College of Computer Science and Technology, Ministry of Education, Jilin University, Changchun, China

**Keywords:** age prediction, methylomic biomarker, regression, support vector regressor, ridge

## Abstract

Aging was a biological process under regulations from both inherited genetic factors and various molecular modifications within cells during the lifespan. Multiple studies demonstrated that the chronological age may be accurately predicted using the methylomic data. This study proposed a three-step feature selection algorithm AgeGuess for the age regression problem. AgeGuess selected 107 methylomic features as the gender-independent age biomarkers and the Support Vector Regressor (SVR) model using these biomarkers achieved 2.0267 in the mean absolute deviation (MAD) compared with the real chronological ages. Another regression algorithm Ridge achieved a slightly better MAD 1.9859 using the same biomarkers. The gender-independent age prediction models may be further improved by establishing two gender-specific models. And it's interesting to observe that there were only two methylation biomarkers shared by the two gender-specific biomarker sets and these two biomarkers were within the two known age-associated biomarker genes CALB1 and KLF14.

## Introduction

Aging is a ubiquitous phenomenon in almost all the multi-cellular organisms (Horn and Schweppe, 2015). It is also a challenging issue concerned by citizens in many countries (Baltes and Smith, 2003; Banister et al., 2012). Evidences were accumulating about that aging is a biological process strictly regulated by epigenetic modifications rather than random events (Fraga and Esteller, 2007; Martino et al., 2011; Schellenberg et al., 2011; Pal and Tyler, 2016). So it's technically reasonable to estimate an individual's biological age through the biomarkers like telomere length (Saeed et al., 2012; Barrett et al., 2013), age-dependent changes in T cell DNA (Zubakov et al., 2010; Ou et al., 2012), and RNA biomarkers (Alvarez and Ballantyne, 2006), etc. Recent studies also demonstrated that DNA methylation levels at certain CpG residues were linearly associated with the biological ages, and may serve well as age biomarkers (Zubakov et al., 2016).

DNA methylation has been implicated to be involved in various aging-associated biological processes (Jones et al., 2015; Field et al., 2018). DNA methylation is a biological process of selectively adding a methyl group to a cytosine to form 5-cytosine facilitated by a DNA methyltransferase (Moore et al., 2013). This epigenetic modification plays an essential role in transcriptional regulation and other biological processes (Vaillancourt et al., 2017; Suzuki et al., 2019). Quite a few age prediction models were proposed based on the methylation biomarkers. Besides clinical application, these models can also be used in forensic investigation (Vidaki and Kayser, 2018; Alsaleh and Haddrill, 2019). Blood and other liquids are one of the most important biological evidences found in the crime scene, so it's necessary to use the whole blood to establish an accurate age prediction model.

The major challenge is finding a subset of methylation features with a good age prediction performance using the methylomic datasets. About half a million methylation features may be generated for one sample by the popular array-based methylome profiling technologies like Illumina HumanMethylation450 BeadChip (450K) (Fernandez-Jimenez et al., 2019). The feature number is much larger than the sample number, and a step of feature selection has to be conducted to avoid the model over-fitting (Feng et al., 2018).

The existing methylome-based age prediction studies explored different feature selection algorithms to find the best age-associated biomarkers. Horvath used the elastic net algorithm to select 353 methylomic features to predict the human ages and the mean absolute error of the predicted age was about 3.6 years (Horvath, 2013). Yi et al. detected three age-related gene fragments from the blood samples of 40 volunteers and used the CpG locus of these fragments to train the age-regression model with a prediction difference of 4 years compared with the real ages (Yi et al., 2015). Hong et al. proposed a linear regression-based age prediction model, which achieved 94.5% in correlation and 3.13 years in the mean absolute deviation (Small et al., 2011) from the chronological ages (Hong et al., 2017). Another study investigated this forensic problem by selecting 23 methylomic features and established a multi-variate regression model with an age prediction deviation of about 4.6 years (Vidaki et al., 2017).

Feature selection algorithm has been utilized in many biomedical research areas. Various biomedical high-throughput data producing technologies were rapidly invented and developed and may produce as many as millions of features per sample (Diao and Vidyashankar, 2013; Ye et al., 2017; Ceglia et al., 2018). But the number of samples collected in a study was usually limited by the difficulty of patient recruitment and the cost of generating the data. So a biomedical big data project usually had a much larger number of features than the number of samples. A feature selection algorithm may significantly reduce the model complexity and the possibility of over-fitting (Le et al., 2017; Ma and Fan, 2017). Feature selection was not only widely used in the bioinformatics problems of genes (Tian et al., 2019), proteins (Liu et al., 2019), and metabolism system (Grissa et al., 2016), but also played an important role in the analysis of biomedical images (Pan et al., 2019) and time series data (Li et al., 2017).

This study proposed a three-step feature selection algorithm, AgeGuess, to find the best age prediction biomarkers using the methylomic profiles. The metrics Maximal Information Coefficient (MIC) was a sensitive correlation measurement (Reshef et al., 2011) and was utilized to remove those methylomic features with small MIC association with ages. The remaining features were recursively eliminated based on the evaluation of a support vector regressor. The last step removed the features iteratively based on an exhaustive screening. Our experimental data demonstrated an improved prediction performance of chronological ages. Gender information was also evaluated in further optimizing the age prediction models.

## Materials and Methods

### Dataset Summary

This study used the methylomic dataset GSE40279, which was publicly available from the database Gene Expression Omnibus (GEO) (Clough and Barrett, 2016). The dataset GSE40279 was profiled using the methylomic platform Illumina HumanMethylation450 BeadChip (accession GPL13534) (Alsaleh and Haddrill, 2019). There were 656 samples with chronological ages in this dataset, and each sample was profiled for 485,577 methylomic resides (Alsaleh and Haddrill, 2019). The methylome was generated using the human whole blood samples, obtained from 426 Caucasians and 230 Hispanics individuals with chronological ages 19–101. As similar to the existing study (Hannum et al., 2013), sex chromosomes were excluded from analysis in this study. So there were 473,034 CpG features left for further analysis.

### Feature Selection Algorithm AgeGuess

Not all of these half-million methylomic features were associated with the aging process and all the existing studies selected a subset of features for building their age prediction models (Horvath, 2013; Yi et al., 2015; Hong et al., 2017; Vidaki et al., 2017). So this study proposed a feature selection algorithm AgeGuess to find a feature subset with the best age prediction performance.

Single-step feature selection algorithm may be roughly grouped as two major types, i.e., filters and wrappers (Suto et al., 2016). A filter evaluated each feature's association with the class labels with the assumption of inter-feature independence and can be easily scaled to a large number of features (Guyon and Elisseeff, 2003; Solorio-Fernández et al., 2016). A wrapper screened a feature subset by a heuristic rule for its classification performance of a user-defined classifier. A wrapper usually outperforms a filter in accuracy with the cost of a high computational complexity (Guyon and Elisseeff, 2003; Solorio-Fernández et al., 2016). In order to fully utilize the advantages of both filters and wrappers, a multi-step feature selection algorithm may significantly reduce the number of features in the first step. Then more sophisticated and slow algorithms may be utilized. The following algorithm AgeGuess was designed based on this rule for the chronological ages.

Firstly, AgeGuess selected 10,000 methylomic features that were highly correlated with the sample label, i.e., chronological age. There were 473,034 methylomic features for each sample in this dataset, and not all these features contributed to the age prediction. The metrics Maximum Information Coefficient (MIC) demonstrated a very sensitive power in detecting linear and non-linear correlations between two variables (Reshef et al., 2011). This study calculated the MIC correlation of each methylated features with the chronological ages, and kept the 10,000 features with the largest MIC values for further analysis.

Then the Recursive Feature Elimination (RFE) strategy was utilized to remove un-related features. The RFE strategy relied on the feature ranking and iteratively removed the *k* least-ranked features. The investigated problem in this study was a regression model, and the Support Vector Regressor (SVR) was used to calculate the metrics to rank the features. The trained SVR model produced a weight vector Feature Importance, and the features were sorted by the descendent order of the weights. This procedure was conducted iteratively until all the features were removed. The feature subset with the best regression performance was returned.

One more redundancy-removal step was conducted to further refine the feature subset obtained in the above step. The iterative exclusion of the feature with the least performance decrease was carried out, which was the same as the backFS strategy in the other studies (Feng et al., 2019; Zhang et al., 2019). The performance was calculated by the 10-fold cross validation strategy.

A good feature selection algorithm tended to select fewer features and to achieve a higher prediction performance. But these two performance metrics usually cannot achieved simultaneously. So this study defined the integrated evaluation index (EI) as the optimization goal. EI was defined as (MAD+FNum/100), where MAD was the mean absolute deviation and FNum was the number of features selected by the feature selection algorithm. This regression performance metrics suggested one more selected feature increased the overall performance by 0.01. And the metrics EI was used to optimize the above-mentioned backFS strategy.

### Performance Evaluation Metrics

This study investigated the age prediction problem using the 656 samples from the platform GEO. Multiple regression performance metrics were used to evaluate how the generated regression model performed. The metrics Mean Absolute Deviation (Small et al., 2011) was the averaged absolute error value between the predicted age and the chronological age (Pan et al., 2019). The Mean Squared Error (MSE) and the squared root version of MSE (RMSE) were another two widely used regression performance metrics (Liu et al., 2019; Thompson et al., 2019). The metrics Goodness of Fit (R2) quantitatively evaluated how well the regression model fitted the data (Chong et al., 2017). These regression metrics were implemented in the package scikit-learn version 0.19.1 of Python version 3.6.4.

## Results

### Optimizing the Proposed Algorithm AgeGuess

The proposed feature selection algorithm AgeGuess selected 10,000 out of the 473,034 methylomic features with the largest MIC coefficients (Reshef et al., 2011) with the chronological ages. AgeGuess hypothesized that the contributions of the excluded features may be neglected since their MIC coefficients with the chronological ages were small.

**Figure 1:**
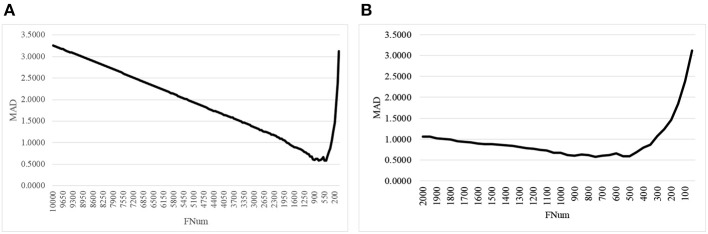
The line plot of the regression metrics MAD of AgeGuess. **(A)** RFE strategy to removed 50 features in each iteration on [10000, 50] and **(B)** The scale was zoomed to [2000, 50]. The horizontal axis was the number of features remained for building the classification model.

The second step of AgeGuess utilized the RFE framework to iteratively remove the features, as shown in Figure 1. Due to the number of remaining features was still very large, this study set *k* = 50, i.e., 50 features with the least Feature Importance weights calculated by the trained SVR model were removed in each iteration. Figure 1A illustrated that the majority of the 10,000 methylation features didn't contribute to the age prediction performance. And there was a “valley” smaller than 1,500 features in the line plot in Figure 1A. So Figure 1B zoomed in the line plot within the range [2000, 50]. The data showed that the small MAD value was achieved between 900 and 500. And the minimum value MAD = 0.5809 was achieved with 750 features.

**Figure 2:**
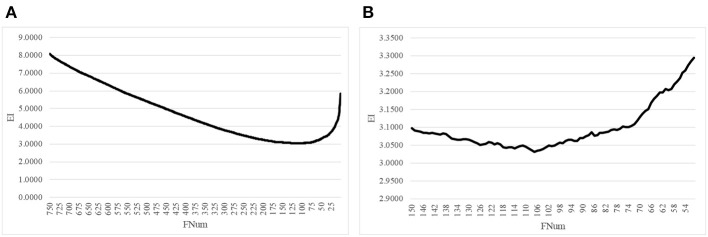
Iterative elimination of redundant features by backFS. **(A)** The line plot for the numbers of features no more than 750 features. **(B)** The zoomed-in plot for the numbers of features between [50, 150]. The horizontal axis was the number of features. And the vertical axis was the regression performance metrics EI.

The proposed algorithm AgeGuess further removed the redundancies in the methylated features by the function backFS (Feng et al., 2019; Zhang et al., 2019). The 750 methylation features chosen in the above step was iteratively evaluated and one feature was removed per iteration if its removal generated the least contribution to the age prediction performance metrics EI. Figure 2A illustrated that the valley was around 100 features in the horizontal axis. The plot was further zoomed-in for the number of features between 50 and 150, as shown in Figure 2B. The age regression metrics EI reached the minimum 3.0316 when 107 features were selected.

**Figure 3:**
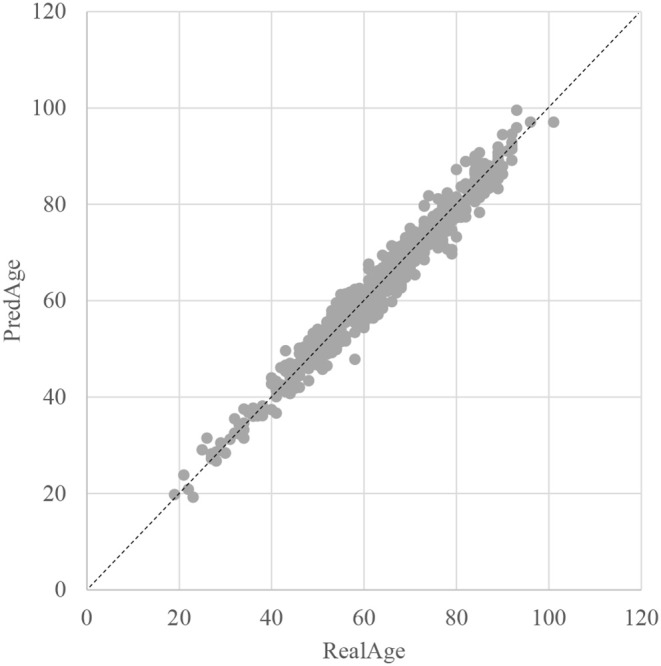
Dot plot between the real chronological age and the predicted age of these samples. The horizontal axis was the chronological age of a sample (RealAge) and the vertical axis was this sample's age averaged over the 10-fold cross validation (PredAge). The regressor was SVR. The perfect prediction of age was represented by the gapped line y=x.

The SVR regression model was trained using the 107 methylation features, and was evaluated by the following regression performance metrics. Figure 3 illustrated that the RealAge and the PredAge were very close to each other. The prediction performance was averaged over the 10-fold cross validations, and 10 random rusns were averaged to generate the final results. The Mean Absolute Deviation (Small et al., 2011) was 2.0267 years. AgeGuess's model achieved the other two metrics RMSE and R2 were 1.6149 and 0.9672, respectively. The regression coefficients of the methylomic features were given in Supplementary Table 1.

### Comparison With Other Commonly Used Feature Selection Algorithms

This study compared the proposed AgeGuess with the existing feature selection algorithms. Three filter algorithms were evaluated, i.e., the uni-variate F-Regression (FR), Mutual Information (MI), and Pearson Correlation Coefficient (PCC). Filter algorithms returned an ordered list of all the features and the same number of features as AgeGuess was used for a fair comparison. Three recursive feature elimination (RFE) algorithms were also compared with AgeGuess, i.e., L1-RFE, L2-RFE, and SVR-RFE. An RFE algorithm eliminated a feature if its removal induced the least regression performance loss. And the regression performances of the above three RFE algorithms were calculated by the L1-regularized, L2-regularized and Support-Vector-based regressors, respectively. The number of selected features was an importance factor of a feature selection algorithm. So we also set the number of features selected by these RFE algorithms to the same as AgeGuess.

**Figure 4:**
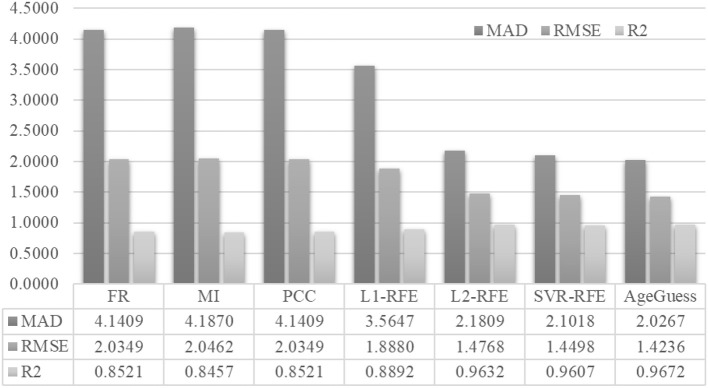
Performance comparison of AgeGuess with six existing feature selection algorithms. The regression performance metrics MAD, RMSE, and R2 were Mean Absolute Error, squared root of mean squared error, and the Goodness of Fit (R2), respectively.

Figure 4 demonstrated that AgeGuess outperformed the existing feature selection algorithms in all the three regression performance metrics. AgeGuess achieved 2.0267 in MAD, which was 2.1142 smaller than that of FR and 2.1603 smaller than that of MI. A larger R2 value suggested that a regressor performed better. AgeGuess achieved the best R2 and outperformed the next best algorithm L2-RFE by 0.0040 in R2. The smaller RMSE was the better. And AgeGuess outperformed the next best algorithm SVR-RFE by 0.0262 in RMSE.

We also compared our best model with the existing age prediction models and AgeGuess performed the best on estimating the chronological ages. Weidner et al. used 102 methylation features from the same dataset as this study to establish their age predictor, which achieved 4.12 in MAD, 5.34 in RMSE and 0.87 in R2 (Weidner et al., 2014). Another study also used the same dataset as this study and detected 41 methylomic features as the age biomarkers. They built the age predictor achieving 10.69 in MAD (Sarac et al., 2017). The same features from the study (Shadrina et al., 2018) were used to train the regressor as in this study and the age predictor only achieved 9.9017 in MAD, 12.1120 in RMSE and 0.0521 in R2, respectively.

### Gender Specificity of Age Prediction

The literature provided different ideas on the correlations between aging and gender variations. Hannum et al. proposed that aging was impacted by various factors and utilized the information of gender and body mass index (BMI) together with the methylomic features in building an age predictor (Hannum et al., 2013). Their model achieved 3.9 years in the age prediction errors and 96% in the correlations of the predicted ages with the chronological ages. Their data suggested that gender was a significant factor to the aging rate. But professor Steve Horvath hypothesized that an age-dependent CpG signatures may be defined independent of genders and his group built a gender-independent age predictor achieving 3.6 years in the metrics median error.

**Figure 5:**
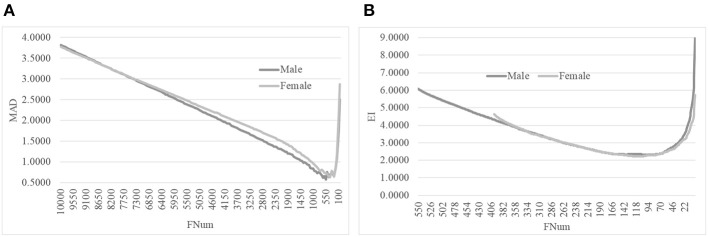
Line plots of AgeGuess's steps 2 and 3. **(A)** The second step of AgeGuess screened features using SVR-RFE. **(B)** The third step of AgeGuess further eliminated redundant features by backFS.

We evaluated this hypothesis with the gender-specific models using the same feature selection algorithm on the same dataset, as shown in Figure 5. The original dataset was split into the dsMale and dsFemale datasets, and the same feature selection procedure AgeGuess was carried out on these two datasets. Figure 5A suggested that AgeGuess achieved 0.5783 and 0.6287 in MAD for the datasets dsMale and dsFemale, respectively. Figure 5B demonstrated that the last step of AgeGuess further refined the gender-specific models to achieve 2.2954 and 2.2148 in EI, respectively. So the Male and Female models outperformed the model using the dataset dsMale∪dsFemale by at least 0.6605 in MAD. And the gender-specific models used the similar numbers of features compared with the original model using the dataset combined from both dsMale and dsFemale.

**Figure 6:**
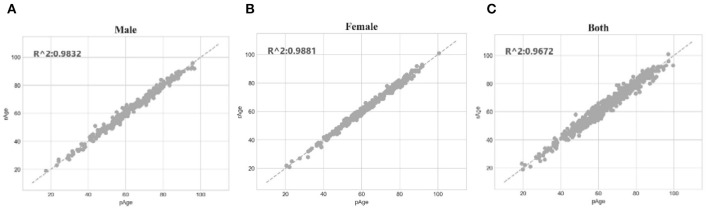
Gender-specific age prediction performances. **(A)** Dot plot for male samples. **(B)** Dot plot for female samples. **(C)** Dot plot for the dataset combined both male and female samples. The perfect prediction of age was represented by the gapped line y=x.

The SVR regression model trained on the dataset dsMale achieved 1.5072 in MAD, 1.3804 in RMSE and 0.9832 in R2. The three performance metrics of the model trained on dsFemale were 1.1669, 1.2112, and 0.9881, respectively. So both gender-specific models outperformed the best model trained over dsFemale∪dsMale, which achieved 2.0267 in MAD, 1.6149 in RMSE and 0.9672 in R2. The dot plots in Figure 6 illustrated how well gender-specific age prediction models achieved on estimating the chronological ages. The regression coefficients of the methylomic features for the two gender-specific models were given in Supplementary Tables 2, 3.

### Evaluating AgeGuess on Another Methylomic Dataset on the EPIC BeadChip

A new methylation probing array, the Infinium MethylationEPIC (EPIC array), was recently launched and provided 868564 methylomic features, which was almost two times as that of the Illumina 450 k array. The EPIC array shared about 94% of the probes in the 450 k array (McEwen et al., 2018; Alsaleh and Haddrill, 2019).

AgeGuess was applied to an independent dataset GSE116339 generated on the EPIC arrays (Curtis et al., 2019). This dataset was publicly available from the database Gene Expression Omnibus (Clough and Barrett, 2016) and provided the methylomes of 679 whole blood samples with the chronological ages (Curtis et al., 2019). AgeGuess finally selected 388 CpG features to establish the age prediction model. Two hundred fourteen of these 388 features were shared with the 450 k array and the other 174 features were EPIC-specific. The Mean Absolute Deviation (MAD) of this model was 2.4780, while the other two metrics RMSE and R2 were 1.8101 and 0.9319, respectively. So the EPIC array-based model performed slightly worse in the metrics MAD than the model based on the 450 k array. And it also used more than three times of features than the 450 k array-based model. The experimental data suggested that the EPIC array may need the 6% of the 450 k array-specific methylomic features to precisely describe the aging process.

### Impact of Training Dataset Sizes on Age Prediction Performances

**Figure 7:**
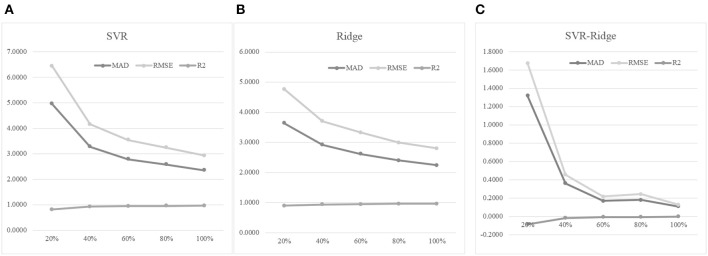
The training dataset size was important for the age prediction performance. **(A)** The regression model was trained using the regressor SVR. **(B)** The regression model was trained using the regressor Ridge. **(C)** The performance metrics of SVR minus those of Ridge. The horizontal axis was the percentage of the training dataset used for training the model. The three regression performance metrics MAD, RMSE and R2 were calculated.

An experiment series was carried out to evaluate how different numbers of training samples may impact the age prediction performances, as shown in Figure 7. Firstly, 30% of the whole dataset was randomly selected as the test dataset. Then we randomly selected 20, 40, 60, 80, and 100% of the remaining samples to train the regression models, and tested the model prediction performances on the test dataset. Figure 7A suggested that more training samples did improve the regression model's performances. The 40% model improved the 20% model by 33.94% in MAD, but the 60% model only achieved a 14.94% improvement in MAD compared with the 40% model. And even smaller improvements were achieved when more training samples were added. Similar patterns were observed for the other two regression performance metrics RMSE and R2.

Another regression algorithm Ridge was evaluated for its age prediction performances using the same features, as shown in Figures 7B,C. The Ridge-based age prediction models also demonstrated a similar pattern on different numbers of training samples, as shown in Figure 7B. After 60% of samples in the training dataset was used to train the model, more training samples didn't facilitate a major model improvement. We calculated the metrics differences between SVR and Ridge, as shown in Figure 7C. A small value of MAD or RMSE suggested a good age prediction model, and Figure 7C illustrated that the MAD or RMSE values of Ridge were always smaller than those of SVR. And a large R2 value suggested a good regression model. Figure 7C illustrated that Ridge was always larger than SVR in the performance metrics R2. So the regression algorithm Ridge outperformed SVR in all the three regression performance metrics MAD, RMSE, and R2.

### The Biological Relevance of Age Biomarkers to the Aging Process

**Figure 8:**
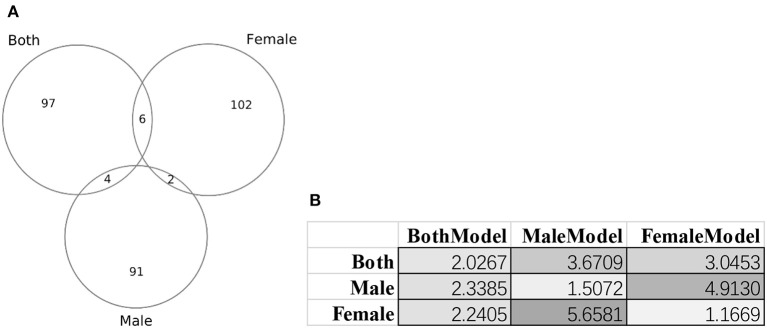
Gender-specific methylomic biomarkers for age prediction. **(A)** Venn plot of the three sets of methylomic biomarkers. The dataset “Both” included both male and female samples. The two datasets “Male” and “Female” consisted of male and female samples, respectively. **(B)** Each column gave the metrics MAD values of the age regression SVR model trained by the biomarker set denoted on the first row. BothModel, MaleModel and FemaleModel denoted the sets of biomarkers detected using the datasets Both, Male, and Female.

Figure 8 illustrated that there were little overlaps between the gender-specific methylomic biomarkers, and there were no methylomic biomarkers shared among the three sets of biomarkers BothModel/MaleModel/FemaleModel. The data suggested that there existed differences in aging biomarkers between males and females. Even the aging biomarkers of the BothModel performed worse on the individual genders (datasets dsMale and dsFemale). And the cross-gender validation demonstrated much worse age regression performances, as shown in Figure 8B.

Some of the gender-specific age methylomic biomarkers were known to have gender-biased expression patterns (Gershoni and Pietrokovski, 2017). There were two female-biased age methylomic biomarkers were cg06419846 (gene CD248) and cg25371036 (gene AMOTL1), which were from the chromosome 11 (Gershoni and Pietrokovski, 2017). CD248 was observed to be hypermethylated during aging and suggested the impaired T cell functionality in the aged adults (Tserel et al., 2015). AMOTL1 (Angiomotin Like 1) was also differentially expressed in different age groups of females, which was verified by the quantitative real-time PCR (qRT-PCR) (Pelissier et al., 2014).

Some of the male-specific age methylomic biomarkers in this study were also supported by the literature. Both of the two biomarkers cg25478614 (gene SST) and cg04084157 (gene VGF) were observed to exhibit male-biased expression patterns (Gershoni and Pietrokovski, 2017). The gene SST received hypermethylation to decline its expressions gradually with age (McKinney et al., 2015). The SST+ neurons may also be impacted with chronic exposures to different photoperiods and resulted in behavioral alternations (Pritchard et al., 2019). The gene VGF encoded the Nerve Growth Factor Inducible protein and gradually increased its expressions in the T lymphocytes when the host age increases (Busse et al., 2014).

These gender-specific biomarker genes were screened by the online GO (Gene Ontology) analysis system DAVID version 6.8 (Huang da et al., 2009a,b). The biomarker genes were input as the foreground and the species Homo sapiens was chosen as the background. The enriched terms with *P* ≤ 0.05 in the functional annotation chart were collected for further analysis, as shown in Supplementary Table 4. Figure 8A suggested that the three datasets dsBoth, dsFemale and dsMale shared very few biomarkers. Supplementary Table 4 further supported the observation with that only one GO term (biological process “regulation of catalytic activity”) was shared by two datasets dsBoth and dsMale. The top two ranked terms in the female biomarkers were two molecular function terms “RNA polymerase II transcription factor activity, ligand-activated sequence-specific DNA binding” and “RNA polymerase II core promoter proximal region sequence-specific DNA binding.” The female-specific aging associated RNA polymerase II activities were supported by the experimental evidences observed from the female rat brain (Shults et al., 2015) and the female rat liver (Spindler et al., 1991). While we focused on the aging biomarkers from the dataset dsBoth, the top-ranked enriched GO term was the biological process “homophilic cell adhesion via plasma membrane adhesion molecules,” as shown in Supplementary Table 4. It is well-known that the growth hormone was actively involved in the aging process and some of the state-of-the-art results were reviewed in Allshouse et al. (2018) and Bartke (2019).

## Discussion

The aging process was impacted by both inherited genetic and environmental factors. Multiple studies demonstrated that the methylomic biomarkers served as a rich information source for predicting the chronological ages (Hong et al., 2017; Shadrina et al., 2018). Most of the existing studies selected their age biomarkers based on these biomarkers' biological relevance to the aging process (Zubakov et al., 2016) or statistically correlations with the chronological ages (Shadrina et al., 2018).

This study hypothesized that the chronological age may be more accurately predicted using delicately chosen methylomic biomarkers. A three-step feature selection algorithm AgeGuess was proposed and evaluated for the age regression problem based on the methylomic features. The SVR model using the AgeGuess-selected methylomic biomarkers outperformed the existing age prediction models. Our experimental data suggested that another regression algorithm Ridge achieved a slightly better age regression performance compared with the SVR model. So the AgeGuess-selected features represented important age biomarkers independent of regression algorithms.

This study further investigated whether the age process was gender-specific. The proposed algorithm AgeGuess selected 97 methylomic biomarkers for the male samples, and 110 biomarkers for the females. But there were only two methylomic biomarkers cg26290632 (gene CALB1) and cg07955995 (gene KLF14) selected by AgeGuess in both the male and females samples. Both CALB1 (Loerch et al., 2008) and KLF14 (Small et al., 2011) were known age-related biomarkers. CALB1 demonstrated robustly down-regulated expression across rhesus monkeys and humans (Loerch et al., 2008; Pabba et al., 2017). While KLF14 served as a master regulator of many genes and its altered methylation patterns were associated with the aging process (Spolnicka et al., 2018). But both of these two genes didn't demonstrate gender-specific patterns. So these two genes may be robust age biomarkers without gender-bias. Some of the gender-specific age methylomic biomarkers were also supported by the literature.

The age prediction models proposed in this study may need further validated by various tissue samples. Gene expression patterns differred across tissues, so did patterns of DNA methylation (Decato et al., 2017; Zhou et al., 2017; Slieker et al., 2018). Only whole blood methylation samples were used in this study. Considering the influence factors such as tissues and environments, the age prediction models in this study may have reduced prediction capabilities for forensic samples other than whole blood. In addition, Hannum et al., demonstrated that some electronic health record (EHR) data like BMI may be integrated with the methylomic data to achieve a better age prediction (Hannum et al., 2013). So more types of biomedical data of the participants may further improve the proposed models.

## Data Availability Statement

Publicly available datasets were analyzed in this study. The EPIC array dataset can be found here: https://www.ncbi.nlm.nih.gov/geo/query/acc.cgi?acc=GSE116339.

## Author Contributions

FZ and XG conceived and designed the project and polished the manuscript. XG, SL, XF, and MD wrote the code and conducted the experiments. XG and HS worked on the manuscript revision according to the reviewers' comments. XG and LH discussed the experimental results and drafted the manuscript. All authors read and approved the final version of the manuscript.

### Conflict of Interest

The authors declare that the research was conducted in the absence of any commercial or financial relationships that could be construed as a potential conflict of interest.
