# Supplementary material for: AgeGuess, a Methylomic Prediction Model for Human Ages
Source: Front Bioeng Biotechnol. 2020 Mar 10;8:80. doi: 10.3389/fbioe.2020.00080 (PMC7075810; doi:10.3389/fbioe.2020.00080)
Supplement: Supplementary file 1 [file Data_Sheet_1.PDF]

AgeGuess, a methylomic prediction model for human ages

Xiaoqian Gao[1], Shuai Liu[1], Haoqiu Song[2,1], Xin Feng[1], Meiyu Duan[1], Lan Huang[3], Fengfeng Zhou[1,#].

1. BioKnow Health Informatics Laboratory Key Laboratory of Symbolic Computation and Knowledge Engineering, College of Computer Science and Technology, Ministry of Education, Jilin University, Changchun 130012, China
2 College of Computer Science, Hubei University of Technology, Wuhan, Hubei 430068, China;
3. Key Laboratory of Symbolic Computation and Knowledge Engineering, College of Computer Science and Technology, Ministry of Education, Jilin University, Changchun 130012, China

# Correspondence: author: Fengfeng Zhou, e-mail: FengfengZhou@gmail.com or ffzhou@jlu.edu.cn . Web site: http://www.healthinformaticslab.org/.

Supplementary Table S1

The SVR coefficients of the CpG features for the age regression model of both genders were given here. Columns “Cpgsite” and “Coef” were the feature names and coefficients, respectively. The other three columns were the functional annotation data collected from the definition file of the methylomic platform Illumina HumanMethylation450 BeadChip (accession GPL13534).

CpGsite	Coef	UCSC_RefGene_Name	UCSC_CpG_Islands_Name	HMM_Island
cg16867657	2.2887	ELOVL2	chr6:11043913-11045206	6:11151611-11153237
cg08097417	1.3055	KLF14	chr7:130417912-130419378	7:130068467-130069793
cg23615741	-1.2522		chr10:101297438-101297703	10:101286334-101287693
cg04940570	1.0538	TEAD1	chr11:12695414-12696981	11:12651991-12653557
cg13612317	-0.4737	KIF5B	chr10:32344550-32345570	
cg02924487	0.9283	TP73;TP73;TP73;TP73	chr1:3649294-3649674	
cg03399905	0.7830	ANKRD34C	chr15:79576059-79576270	15:77363046-77363443
cg00583733	-1.3004	ALDOA;ALDOA;ALDOA	chr16:30076310-30077872	
cg01872593	-0.8526			
cg02650266	1.5072		chr4:147558231-147558583	4:147777501-147778016
cg05274755	-0.9258	NPAS3;NPAS3;NPAS3;NPAS3		14:32476987-32478561
cg10370262	0.7718	PRKAG2;PRKAG2		
cg26795340	0.9920	GPD1;C12orf62	chr12:50505138-50506235	
cg10493324	-0.9206		chr11:118977878-118978785	
cg27184903	-0.7133	APBA2;APBA2		
cg17168836	-0.8758	GNG12		
cg14566959	0.9993	PCDHGA4;PCDHGA6;PCDHGA1;PCDHGA5;PCDHGB1;PCDHGB4;PCDHGA3;PCDHGA2;PCDHGA8;PCDHGA7;PCDHGB2;PCDHGA8;PCDHGB3	chr5:140772385-140772682	5:140752570-140752866
cg07318287	-1.6007	IL6R;IL6R	chr1:154377670-154378445	
cg00417421	-0.7369	TSHZ2;TSHZ2	chr20:51589707-51590020	20:51023023-51023393

cg14871932	0.7125	GCK;GCK;GCK	chr7:44184770-44185695	7:44151296-44153080
cg06365016	0.7148	PIWIL1	chr12:130822360-130822696	12:129387406-129387612
cg05826458	-0.6284	ACOXL	chr2:111875206-111880965	2:111591016-111592221
cg01074797	-0.9828	PDZK1IP1		
cg23344321	0.6141	CPD	chr17:28705363-28706818	
cg27432653	0.6141	GFI1;GFI1	chr1:92945907-92952609	1:92724308-92725197
cg14614094	0.8884	EXOSC2	chr9:133569082-133569451	9:132557429-132557909
cg18433086	0.9261	ABL2;ABL2;ABL2;ABL2;ABL2	chr1:179198163-179199200	
cg19414591	-0.9462		chr1:91190489-91192804	1:90963042-90963258
cg01528542	-0.6734		chr12:81471569-81472119	
cg26483332	-1.1384	COMTD1	chr10:76993892-76995953	
cg12096762	-0.8747			
cg07336350	0.8561		chr16:54317821-54324604	16:52879155-52879917
cg10981464	0.5667	NTRK1;NTRK1;NTRK1	chr1:156828804-156831102	1:155095438-155097726
cg05061107	0.7618	SF1;SF1;SF1	chr11:64532936-64533763	11:64289451-64289773
cg08330117	0.9419	RIPK2	chr8:90769535-90770457	
cg25401628	0.8553	SULF2;SULF2;SULF2	chr20:46413742-46415507	
cg24756391	0.4127	BCL2L13	chr22:18121280-18122009	22:16501065-16501976
cg03605567	0.5153		chr8:991532-991862	8:978685-979306
cg02899788	-1.1403	ATPAF1;ATPAF1	chr1:47133674-47134395	
cg09651136	-0.5910	PKM2;PKM2;PKM2	chr15:72522131-72524238	
cg04410989	-1.0034	SAMHD1	chr20:35579337-35580451	
cg01329511	0.6743	SMYD5	chr2:73441313-73441817	
cg01877778	0.7104	PTPRN2;PTPRN2;PTPRN2	chr7:157413901-157414146	7:157107694-157108425
cg16185666	0.6219	WDFY2	chr13:52158056-52159235	
cg05203217	-0.8313	KCNS1	chr20:43729515-43729791	
cg11806672	0.6193	POU4F1	chr13:79175610-79177985	13:78073612-78074696
cg18406708	-0.5998	FAM82A1;FAM82A1;FAM82A1;FAM82A1		

cg09848445	0.5124	RET;RET	chr10:43600146-43601165	10:42921846-42922001
cg27195326	-0.5756	CNTN1;CNTN1	chr12:41086522-41087102	12:39373726-39373852
cg10891482	0.9128	MS4A8B;MS4A8B		
cg13806741	0.4542	FAM181B;FAM181B	chr11:82443435-82444976	11:82121084-82122721
cg05336268	0.6059	KIAA0556		16:27695674-27695774
cg17105814	-0.4990			
cg12160664	0.6092	OLFML3		
cg07010228	-0.4191	JSRP1	chr19:2250560-2253959	19:2201561-2205121
cg00940560	0.5913	MBOAT4		
cg08708629	-0.8668	PDE4DIP		
cg26019680	0.7042	PODXL2	chr3:127347682-127348859	3:128830434-128831529
cg02206259	0.5154	C1orf198;C1orf198	chr1:231003631-231004655	
cg09028365	0.4497	EPHA8;EPHA8	chr1:22889595-22890096	
cg13221458	-0.5557	SOD2;SOD2;SOD2	chr6:160113557-160114863	
cg23628684	0.8839	CCT8	chr21:30445575-30446255	21:29367413-29368268
cg01863374	-0.7155		chr17:66197027-66197278	17:63708112-63708839
cg26851782	-0.4568	GLRA1;GLRA1	chr5:151304226-151304824	
cg25026401	0.6978	SLC15A4	chr12:129307947-129308724	12:127873876-127874639
cg03637614	-1.2242	ZC3HC1	chr7:129690960-129691326	7:129478122-129478562
cg10983206	-0.6101			10:7604407-7604477
cg16179607	-0.5096	PPP3R1	chr2:68479061-68480040	
cg16347361	0.7378	TIMP2	chr17:76851759-76851973	
cg20010135	-0.7205	HSD3B7;HSD3B7;HSD3B7		
cg21751873	0.3801			
cg07927379	0.6349	C7orf13;RNF32	chr7:156432433-156433670	7:156125195-156126707
cg09884851	-0.5744	SYT1;SYT1;SYT1		
cg00903584	0.5662	PTPN7;PTPN7;PTPN7		
cg11143148	0.4750	TTC7B	chr14:91282227-91282757	
cg14289461	0.5772	LAMA4;LAMA4;LAMA4;LAMA4;LAMA4	chr6:112575091-112575483	
cg06828936	-0.6291	DPP10;DPP10	chr2:115918737-115920765	

cg04451175	-0.5601	FGF20	chr8:16859044-16859452	
cg21159778	0.5172	DFNB31;DFNB31	chr9:117266468-117268094	9:116306326-116307866
cg02513556	-0.3718			12:9987169-9987420
cg01146324	0.3714			
cg00328058	-0.7093	PLEKHN1;KLHL17;PLEKHN1	chr1:894313-902654	1:890608-890926
cg19401340	0.3626	PPM1E	chr17:56832961-56833986	17:54187961-54189321
cg24338748	0.5253	DHRS11	chr17:34948053-34948902	17:32021950-32022974
cg23860263	-0.7391	RNF34;RNF34	chr12:121837380-121838396	12:120321774-120322985
cg16905506	-0.4519	EFNA4;EFNA4;EFNA4	chr1:155034925-155036578	1:153301550-153302508
cg08375754	-1.0327	TTC9B	chr19:40723104-40724389	19:45415669-45416229
cg09407429	1.0915	ITPR1;ITPR1;ITPR1	chr3:4534412-4535367	3:4508938-4510367
cg18440889	0.5427	SHANK2		
cg00486113	-0.4491	PSORS1C1;PSORS1C2		
cg02340556	0.3896		chr17:46796234-46797292	
cg23669043	-0.4398	CTSF	chr11:66335575-66336151	
cg11700800	0.5933	FLJ42709;FLJ42709;FLJ42709	chr5:92906239-92908875	5:92931903-92933043
cg18816996	0.4998	COL23A1	chr5:178016558-178017670	
cg19283806	-0.6676	CCDC102B		
cg01993411	-0.4584	PCIF1	chr20:44562892-44564220	20:43996120-43997492
cg23861078	0.7844	SLC5A6;C2orf28;SLC5A6;C2orf28;C2orf28	chr2:27434432-27435720	2:27289134-27289224
cg00398048	0.4969	AGA	chr4:178363239-178363708	
cg26659569	0.4113	TMCO3		13:113227898-113228464
cg27569300	0.3742	SYNM;SYNM	chr15:99645030-99646444	15:97462554-97464153
cg04793090	0.6324	CNBP;CNBP;CNBP;CNBP;CNBP;CNBP	chr3:128902134-128902978	
cg23386236	-0.3389	CAPN3;CAPN3;CAPN3;CAPN3;CAPN3;CAPN3		

cg09594708	0.5213	PPP1R3E	chr14:23770512-23771902	14:22840266-22842007
cg14474150	0.4007	CDC42BPB	chr14:103430534-103430787	14:102502824-102503151
cg25236427	-0.5326	CCDC52	chr3:113233663-113234094	
cg23152772	-0.3183	FIBCD1;FIBCD1	chr9:133813631-133815781	
cg26714410	0.7203	TSC22D1	chr13:45149970-45152288	13:44047944-44050460

Supplementary Table S2

The SVR coefficients of the CpG features for the age regression model of female samples were given here. Columns “Cpgsite” and “Coef” were the feature names and coefficients, respectively. The other three columns were the functional annotation data collected from the definition file of the methylomic platform Illumina HumanMethylation450 BeadChip (accession GPL13534).

CpGsite	Coef	UCSC_RefGene_Name	UCSC_CpG_Islands_Name	HMM_Island
cg16867657	0.9335	ELOVL2	chr6:11043913-11045206	6:11151611-11153237
cg10501210	-0.9590			1:206063625-206063801
cg03399905	0.6204	ANKRD34C	chr15:79576059-79576270	15:77363046-77363443
cg07955995	0.9326	KLF14	chr7:130417912-130419378	7:130068467-130069793
cg25371036	-0.5580	AMOTL1	chr11:94501366-94502696	
cg16181396	0.4489	ZIC1	chr3:147126988-147128999	3:148608809-148608897
cg06231995	0.5373		chr9:140023747-140025010	9:139143349-139144831
cg04558362	-0.6881	C11orf2	chr11:64863698-64863980	11:64619714-64620804
cg27230067	0.7445	AK5;AK5		
cg15802091	0.3511	HSPA2	chr14:65006874-65009197	14:64076491-64079226

cg01528542	-0.6136		chr12:81471569-81472119	
cg05308819	-1.4259			
cg18548400	0.6533		chr3:182510866-182512282	
cg26353783	0.5044	GATAD2B	chr1:153894837-153895921	
cg22254135	-0.4506	CCL16		
cg21922223	-0.9060			
cg26035489	-0.5762	ZMAT5;UCRC;UCRC;ZMAT5	chr22:30162836-30163532	
cg00748589	0.8633		chr12:11653232-11653775	12:11544500-11545229
cg21756476	0.8972	RORA		
cg16054275	-0.6283	F5		
cg05066503	0.3541	WDR27	chr6:169977218-169977894	6:169720073-169720338
cg17817474	0.5107	NGRN;NGRN	chr15:90808574-90809251	15:88610378-88610634
cg03259243	0.9777		chr17:21355954-21356418	17:21296383-21296688
cg01074797	-0.7929	PDZK1IP1		
cg05887079	-0.5690	CMIP;CMIP		16:80163383-80163597
cg16636935	-0.7827	TPI1;TPI1;TPI1;TPI1	chr12:6976392-6977393	12:6846631-6847672
cg05447486	0.7506	RAP1A;RAP1A	chr1:112162204-112162837	1:111963706-111964503
cg10850863	0.9646	MYO18A;MYO18A	chr17:27503598-27504014	
cg19191984	-0.9100	PDE5A;PDE5A;PDE5A	chr4:120548203-120550250	4:120769004-120769900
cg12225226	0.4777		chr13:79170114-79171231	13:78066313-78066421
cg06419846	0.5012	CD248	chr11:66083572-66083782	11:65840060-65841164
cg27091422	-0.5033		chr4:30718853-30719847	

cg09882526	-0.8365	PMS2L4;STAG3L4	chr7:66767145-66768031	7:66404594-66405450
cg25374813	0.7083	SLC23A1;SLC23A1		
cg21186299	0.6178	VGF;VGF	chr7:100806279-100809064	7:100594926-100596772
cg17731209	-0.5776		chr21:38935854-38936546	
cg13251666	-0.3926			
cg18318878	0.3177		chr1:214158726-214159080	1:212225090-212225703
cg24774812	-0.4984	RHOJ		
cg23500537	0.6386			5:140400003-140400154
cg20056341	0.7697	GALK2;GALK2		15:47344893-47345057
cg00584000	0.4241	TCHH	chr1:152084994-152085215	1:150348658-150352050
cg22575379	-0.3322	NUDT16P;NUDT16P	chr3:131080578-131081191	
cg21662478	-0.4994		chr10:42737286-42739108	10:42058403-42059144
cg24772267	0.7568	C6orf155	chr6:72129508-72130756	6:72186063-72187521
cg08841952	0.6395	ERCC1;ERCC1;ERCC1	chr19:45926661-45927191	19:50619344-50619435
cg05826458	-0.6366	ACOXL	chr2:111875206-111880965	2:111591016-111592221
cg24308336	0.6773	C20orf95	chr20:37274691-37275134	
cg19401340	0.3265	PPM1E	chr17:56832961-56833986	17:54187961-54189321
cg01606885	-0.5261			
cg04689379	-0.6078	GIPR	chr19:46174372-46174575	
cg08223225	0.5233	FHIT;FHIT		
cg26129669	-0.5737	GABBR1;GABBR1;GABBR1	chr6:29595298-29595795	
cg05825121	-0.6302			
cg01882933	0.5990	BTNL9		
cg22639561	0.8884			

cg18853490	-0.5590	CHRM3		
cg00548708	0.5921		chr17:48041282-48043064	17:45396167-45396750
cg04521765	-0.6875	LOXL4	chr10:100028204-100028508	
cg12581229	-0.4629	ZNF329	chr19:58661737-58662287	19:63353458-63354099
cg26921612	-0.4195	DEPDC5;DEPDC5;DEPDC5	chr22:32149762-32150064	
cg24414127	0.9388	KBTBD11	chr8:1921559-1922872	
cg27075187	0.4423	TEAD1;TEAD1	chr11:12695414-12696981	11:12651991-12653557
cg16544989	0.4909			16:84157503-84158319
cg16930811	0.3565	CAMKV	chr3:49906737-49907482	
cg00846400	-0.4887	RBM9;RBM9	chr22:36424219-36425303	22:34754065-34755313
cg07412317	-0.3734			16:13890555-13890964
cg14897298	0.5392	ATG2B		14:95820234-95820289
cg26615224	-0.4301	TCF3;TCF3	chr19:1621666-1621901	
cg02960948	0.7163	SMURF2	chr17:62657409-62658858	
cg25389463	-0.3692	RBM34;RBM34;RBM34	chr1:235324264-235324717	
cg22040301	-0.5634	SLC22A18AS;SLC22A18	chr11:2923301-2923817	
cg22257747	0.5826			6:27868552-27868790
cg22891191	-0.3974	PTPRE	chr10:129705217-129706237	10:129595148-129596249
cg11791350	-0.6195		chr17:881733-883381	17:832050-832184
cg27598208	0.6979	TCEA2	chr20:62688551-62688878	
cg00753885	-0.4894		chr12:57399868-57400226	
cg16867680	-0.2973			
cg19542841	-0.5342	ESRRA	chr11:64072064-64073914	11:63828526-63830490

cg24794107	0.4104	SLC39A7;RXRB; SLC39A7	chr6:33167885-33168715	
cg02296167	0.4579	ZGPAT;ZGPAT; ZGPAT	chr20:62366249-62366535	20:61833920-61834335
cg08160331	0.4345	KLHL35	chr11:75140737-75141849	11:74818386-74819728
cg20426994	0.4705	KLF14	chr7:130417912-130419378	7:130068467-130069793
cg06885782	-0.5065	KCNQ4;KCNQ4	chr1:41249090-41250161	
cg07181952	0.3988	ACAP3	chr1:1242400-1245185	
cg26137276	-0.5130		chr6:84140412-84140998	
cg07437988	-0.4359	BCAR4;BCAR4		
cg08719608	0.4836	LOC389333	chr5:138727710-138730911	5:138755676-138758786
cg04635736	-0.6072	FGF11	chr17:7342829-7344028	17:7283616-7284752
cg12869949	0.3990			5:3169575-3169811
cg11770325	0.4215	NAGS;PYY	chr17:42082027-42084972	
cg12329216	-0.3767			
cg17386710	0.2816	AUTS2;AUTS2	chr7:70254894-70255986	7:69890214-69890283
cg13859324	-0.5064	UNC45B;UNC45B		
cg26290632	0.6051	CALB1		8:91163987-91164262
ch.20.24070907R	-0.5533			
cg08302650	0.4179	LRRC24;LRRC14	chr8:145749855-145750410	8:145720626-145721218
cg03271487	0.3297			
cg01804382	-0.4602	TNFRSF21	chr6:47276455-47277823	
cg24551459	0.2856	RAB5C;RAB5C	chr17:40306909-40307263	
cg21874213	-0.5168	NRG2;NRG2; NRG2;NRG2	chr5:139227605-139228279	

cg22617819	-0.3646	ST3GAL3;ST3GAL3;ST3GAL3;ST3GAL3;ST3GAL3;ST3GAL3;ST3GAL3;ST3GAL3;ST3GAL3;ST3GAL3		
cg23934731	0.1967		chr15:89904822-89906050	15:87705827-87707042
cg18488745	0.2795	LOC100130987;CLCF1;CLCF1	chr11:67139887-67141146	11:66897808-66898073
cg24519459	0.2102	BEST2	chr19:12868396-12868840	19:12729429-12729868
cg12930920	0.2593	DIS3;DIS3;PIBF1;PIBF1	chr13:73355703-73356612	13:72253611-72254593
cg03523785	0.3198	FOXG1	chr14:29234889-29235908	14:28304655-28304733
cg22016779	-0.2654	DNER		
cg22353329	0.3321	CBX4	chr17:77812991-77819081	17:75427098-75430838
cg07996594	0.3815	RUNX3;RUNX3	chr1:25255527-25259005	1:25127743-25131723

Supplementary Table S3

The SVR coefficients of the CpG features for the age regression model of male samples were given here. Columns “CpGsite” and “Coef” were the feature names and coefficients, respectively. The other three columns were the functional annotation data collected from the definition file of the methylomic platform Illumina HumanMethylation450 BeadChip (accession GPL13534).

CpGsite	Coef	UCSC_RefGene_Name	UCSC_CpG_Islands_Name	HMM_Island
cg14692377	1.0573	SLC6A4;SLC6A4	chr17:28562387-28563186	17:25586344-25587312
cg07955995	1.6289	KLF14	chr7:130417912-130419378	7:130068467-130069793
cg12580096	-0.9419	C19orf57;CC2D1A	chr19:14016665-14017435	
cg05412028	-0.8927	ABCC4;ABCC4	chr13:95953337-95954211	13:94750898-94750939
cg18939241	0.7879	ATP10A	chr15:26107503-26108818	

cg26290632	0.7619	CALB1		8:91163987-91164262
cg19711579	0.5447	NEUROD1	chr2:182547873-182549177	2:182253454-182254881
cg00486113	-0.4237	PSORS1C1;PSORS1C2		
cg25987082	0.7027	PRPSAP2	chr17:18761273-18761853	
cg25968437	-0.9502			
cg13702357	-0.7150	HIP1	chr7:75368062-75369078	
cg03473532	-1.0055	MKLN1	chr7:131012460-131013190	
cg00443307	0.9482	KLRG1		
cg20359994	0.8547		chr6:27598687-27599146	6:27706677-27707242
cg23916205	0.3435	GATA4		8:11651739-11652008
cg00533891	-0.5615	ZMIZ1		
cg02924487	0.5841	TP73;TP73; TP73;TP73	chr1:3649294-3649674	
cg14975410	0.6087		chr3:171177829-171178964	
cg06544310	-0.7848	HNRNPUL1	chr19:41769215-41769417	
cg16193278	-0.6682	UBAC2;MIR623; UBAC2;UBAC2		
cg11136562	-0.7417	ARAP3		
cg23926526	-0.7319	MDM1;MDM1	chr12:68725847-68726475	
cg08701604	-0.6257	SCNN1B	chr16:23313239-23313946	
cg01436254	0.6052	CD86;CD86		
cg22736354	0.8207	NHLRC1	chr6:18122250-18122994	6:18230230-18231229
cg20554277	0.5907	SLC25A21;SLC25A21		
cg22118131	-0.7382	TBC1D2B;TBC1D2B	chr15:78369517-78369906	15:76156477-76157303
cg02035018	0.7107	ZFYVE28		4:2268958-2270514
cg27569300	0.9383	SYNM;SYNM	chr15:99645030-99646444	15:97462554-97464153
cg22239534	0.6806		chr22:37099471-37099711	
cg24622589	-0.4485			
cg01188578	-0.4513	HADHA	chr2:26467102-26467946	
cg18022322	-0.5216	PCNX	chr14:71373964-71375402	14:70443641-70445200
cg15838173	-0.3776	FUT11	chr10:75531970-75532817	10:75203356-75203438
cg14471619	0.6099	IL15;IL15;IL15	chr4:142557388-142558323	4:142777579-142777958

cg13697715	0.5131	LOC202181	chr5:177098634-177099525	
cg10317380	0.7872	HSPA8;HSPA8	chr11:122932173-122933803	
cg02386604	-0.5219	GOLSYN;GOLSYN;GOLSYN;GOLSYN;GOLSYN;GOLSYN;GOLSYN;GOLSYN;GOLSYN;GOLSYN;GOLSYN;GOLSYN;GOLSYN;GOLSYN;GOLSYN	chr8:110656795-110657291	
cg23409075	-0.6648	DECR1	chr8:91013141-91014266	
cg18050715	-0.5924	MBNL2;MBNL2	chr13:97998962-97999377	
cg02625638	0.3476	FAM83A;FAM83A	chr8:124219411-124219809	8:124287747-124287858
cg11905061	-0.5346	AGAP1;AGAP1	chr2:236578080-236580153	
cg04084157	0.6604	VGF	chr7:100806279-100809064	7:100594926-100596772
cg00163199	-0.6946	ALKBH5	chr17:18086258-18088360	
cg17757602	0.4952		chr5:42951076-42952410	5:42986618-42988127
cg24055029	0.3672	TNXB	chr6:32055067-32055601	6:32163057-32163690
cg02886208	0.4314	SPON1	chr11:14280741-14281164	11:14237354-14237740
cg09651654	0.6043		chr12:7781003-7781248	12:7672271-7672723
cg27401539	0.6438	FAM82A2	chr15:41047210-41047687	
cg07392740	0.4883	KCTD2		
cg08154627	0.2786	CORO2B		
cg06669374	-0.5536			4:185198214-185198434
cg24301724	0.5019	GPR133		12:130182978-130183077
cg00039326	-0.4574	TRAPPC3	chr1:36614855-36615462	
cg11830800	-0.6644	CNTN1;CNTN1	chr12:41086522-41087102	12:39373726-39373852
cg22389438	0.4819			
cg02970836	0.5060		chr16:3238805-3239492	16:3177874-3178032
cg05442902	-0.4718	MGC16703;P2RX6;P2RX6	chr22:21368197-21368771	

cg15062725	-0.5408	ZFR2	chr19:3831363-3831807	19:3781888-3782747
cg13675957	-0.6576	KRT15		
cg11237573	-0.4457	KPNA6	chr1:32574061-32574318	
cg20761322	0.6865	CIB2;CIB2	chr15:78423593-78424096	15:76210313-76211215
cg13367406	0.5196	SLFNL1;SLFNL1	chr1:41483418-41483693	
cg16295725	0.4813	ZNF518B	chr4:10458129-10459353	4:10067138-10068870
cg18506678	0.4316		chr6:85482569-85484718	6:85539303-85539666
cg17714861	0.4737	PRKAG2;PRKAG2	chr7:151421668-151422157	7:151054558-151054673
cg19324997	0.6441	HDAC4		2:239833965-239834218
cg02635865	0.5565	RAB22A;PPP4R1L	chr20:56884738-56885222	20:56317199-56318580
cg09026360	-0.4193	TMIE	chr3:46742764-46743086	
cg03467555	0.3796			16:52961851-52961910
cg00898374	-0.3684		chr13:112691292-112691533	
cg00573770	-0.7666	ZEB2;ZEB2;ZEB2	chr2:145281736-145282269	
cg24517323	-0.3875	NEURL4;NEURL4	chr17:7227416-7227698	17:7168141-7168422
cg07632946	0.3889	BRD8;BRD8; BRD8;KIF20A	chr5:137514660-137514941	
cg20765441	-0.3223	SLC17A2		
cg06171420	0.3040		chr21:47057462-47057820	
cg25478614	0.5078	SST	chr3:187387914-187388176	3:188870501-188870889
cg05086282	-0.5488	PPP2R5C;PPP2R5C	chr14:102227441-102228854	
cg00903584	0.3028	PTPN7;PTPN7; PTPN7		
cg25874421	0.4649	KCNK13	chr14:90526696-90528951	14:89596392-89598737
cg09499629	0.6126	KLF14	chr7:130417912-130419378	7:130068467-130069793
cg03578951	0.3227	DMAP1;DMAP1; DMAP1	chr1:44679003-44679465	
cg11421768	0.3188	TRIP13	chr5:912494-912861	5:965433-965841

cg02741882	0.3949	SEZ6L2;SEZ6L2; SEZ6L2;SEZ6L2	chr16:29887788-29888316	16:29795162-29795791
cg08415592	-0.7221	APOL1;APOL1; APOL1;APOL1		
cg03196381	-0.3775	SELS;SELS	chr15:101817246-101817915	15:99635496-99635618
cg17216759	0.2703	CD47;CD47;CD47	chr3:107809375-107810543	3:109291899-109293252
cg02649547	0.2371	PLCE1;PLCE1		
cg14010720	-0.4335	PTK6	chr20:62168432-62168684	20:61638877-61639906
cg14627089	0.2490		chr19:57149423-57149631	
cg20699549	0.3375	GANC	chr15:42566198-42566404	
cg23091758	0.3044	NRIP3	chr11:9025095-9026315	11:8981699-8983012
cg07056567	-0.4246	GNB2	chr7:100270875-100274003	7:100108817-100111939
cg02006203	0.3524	UPP1;UPP1	chr7:48128046-48128499	7:48094572-48096055
cg09417849	0.3298	ARHGDIG;PDIA2	chr16:332514-332760	
cg00943382	-0.1123			7:156478759-156479117
cg25441737	0.1977	HAAO		

Supplementary Table S4

Gene Ontology (GO) terms enriched in the biomarker genes detected in the datasets dsBoth, dsFemale and dsMale. Column “Gender” gave the dataset where the enriched GO term was detected. The columns “Category” and “Term” were the GO category, and the specific GO term. The column “Pvalue” gave the statistical association significance with the phenotype.

Gender	Category	Term	Pvalue
dsBoth	Biological Process	homophilic cell adhesion via plasma membrane adhesion molecules	2.30E-11
dsBoth	Molecular Function	calcium ion binding	8.40E-08
dsBoth	Cellular Componenet	plasma membrane	2.60E-04
dsBoth	Biological Process	peptidyl-tyrosine phosphorylation	1.10E-03

dsBoth	Biological Process	regulation of cell adhesion	1.30E-03
dsBoth	Biological Process	innervation	3.70E-03
dsBoth	Biological Process	negative regulation of neuron apoptotic process	4.50E-03
dsBoth	Biological Process	positive regulation of neuron projection development	1.00E-02
dsBoth	Cellular Componenet	calcineurin complex	2.00E-02
dsBoth	Cellular Componenet	neuronal cell body	2.20E-02
dsBoth	Cellular Componenet	neuron projection	3.20E-02
dsBoth	Biological Process	positive regulation of vesicle fusion	3.50E-02
dsBoth	Biological Process	regulation of glycolytic process	3.50E-02
dsBoth	Molecular Function	GPI-linked ephrin receptor activity	3.50E-02
dsBoth	Biological Process	protein homotetramerization	3.70E-02
dsBoth	Biological Process	regulation of catalytic activity	4.40E-02
dsBoth	Biological Process	axon guidance	4.70E-02
dsFemale	Molecular Function	RNA polymerase II transcription factor activity, ligand-activated sequence-specific DNA binding	9.00E-03
dsFemale	Molecular Function	RNA polymerase II core promoter proximal region sequence-specific DNA binding	1.30E-02
dsFemale	Biological Process	steroid hormone mediated signaling pathway	2.10E-02
dsFemale	Biological Process	transcription initiation from RNA polymerase II promoter	2.10E-02
dsFemale	Molecular Function	steroid hormone receptor activity	2.10E-02
dsFemale	Molecular Function	growth factor activity	2.70E-02
dsFemale	Biological Process	regulation of macrophage activation	3.40E-02
dsFemale	Molecular Function	transcription factor activity, sequence-specific DNA binding	3.60E-02
dsMale	Biological Process	response to drug	4.60E-05
dsMale	Molecular Function	protein binding	4.20E-03
dsMale	Cellular Componenet	ruffle	5.40E-03
dsMale	Biological Process	tyrosine phosphorylation of Stat5 protein	1.70E-02
dsMale	Molecular Function	protein kinase binding	2.20E-02
dsMale	Cellular Componenet	presynapse	2.70E-02

dsMale	Biological Process	intestinal epithelial cell differentiation	3.00E-02
dsMale	Biological Process	cytoskeleton organization	3.20E-02
dsMale	Biological Process	regulation of catalytic activity	3.30E-02
